# Lipopolysaccharide Enhances Tanshinone Biosynthesis via a Ca^2+^-Dependent Manner in *Salvia miltiorrhiza* Hairy Roots

**DOI:** 10.3390/ijms21249576

**Published:** 2020-12-16

**Authors:** Bin Zhang, Xueying Li, Xiuhong Li, Zhigang Lu, Xiaona Cai, Qing Ou Yang, Pengda Ma, Juane Dong

**Affiliations:** 1 College of Life Sciences, Northwest A&F University, No.3 Taicheng Road, Yangling 712100, China; zhangbin351@nwsuaf.edu.cn (B.Z.); xy_li@nwafu.edu.cn (X.L.); luzhigang@nwafu.edu.cn (Z.L.); yangqingou@nwafu.edu.cn (Q.O.Y.); pengdama@nwafu.edu.cn (P.M.); 2 College of Forestry, Northwest A&F University, No.3 Taicheng Road, Yangling 712100, China; lixiuhong@nwsuaf.edu.cn; 3 College of Innovation and Experiment, Northwest A&F University, No.3 Taicheng Road, Yangling 712100, China; xncai@nwafu.edu.cn

**Keywords:** lipopolysaccharide, tanshinones, Ca^2+^ signaling, *Sm*WRKY

## Abstract

Tanshinones, the major bioactive components in *Salvia miltiorrhiza* Bunge (Danshen), are synthesized via the mevalonic acid (MVA) pathway or the 2-C-methyl-D-erythritol-4-phosphate (MEP) pathway and the downstream biosynthesis pathway. In this study, the bacterial component lipopolysaccharide (LPS) was utilized as a novel elicitor to induce the wild type hairy roots of *S. miltiorrhiza.* HPLC analysis revealed that LPS treatment resulted in a significant accumulation of cryptotanshinone (CT) and dihydrotanshinone I (DTI). qRT-PCR analysis confirmed that biosynthesis genes such as *SmAACT* and *SmHMGS* from the MVA pathway, *SmDXS* and *SmHDR* from the MEP pathway, and *SmCPS*, *SmKSL* and *SmCYP76AH1* from the downstream pathway were markedly upregulated by LPS in a time-dependent manner. Furthermore, transcription factors *Sm*WRKY1 and *Sm*WRKY2, which can activate the expression of *SmDXR*, *SmDXS* and *SmCPS*, were also increased by LPS. Since Ca^2+^ signaling is essential for the LPS-triggered immune response, Ca^2+^ channel blocker LaCl_3_ and CaM antagonist W-7 were used to investigate the role of Ca^2+^ signaling in tanshinone biosynthesis. HPLC analysis demonstrated that both LaCl_3_ and W-7 diminished LPS-induced tanshinone accumulation. The downstream biosynthesis genes including *SmCPS* and *SmCYP76AH1* were especially regulated by Ca^2+^ signaling. To summarize, LPS enhances tanshinone biosynthesis through *Sm*WRKY1- and *Sm*WRKY2-regulated pathways relying on Ca^2+^ signaling. Ca^2+^ signal transduction plays a key role in regulating tanshinone biosynthesis in *S. miltiorrhiza*.

## 1. Introduction

*Salvia miltiorrhiza* Bunge, also known as danshen, is a widely used Chinese herbal medicine for treating cardiovascular and cerebrovascular diseases [1]. The primary bioactive ingredients of *S. miltiorrhiza* comprises salvianolic acids and tanshinones. In these secondary metabolites, tanshinones have received extensive attention for their multiple pharmacological properties, including cardioprotective effects, antitumor activity, anti-inflammatory activity and antibacterial activity [1,2]. The valuable tanshinones consist of tanshinone I (TI), tanshinone IIA (TIIA), tanshinone IIB (TIIB), cryptotanshinone (CT), dihydrotanshinone I (DTI), etc.

In *S. miltiorrhiza,* tanshinone biosynthesis experiences a complicated process. Based on metabonomics and genomics research, isopentenyl diphosphate (IPP) and dimethylallyl diphosphate (DMAPP) have been identified as the precursors of tanshinones. These two compounds can be generated either from the mevalonic acid (MVA) pathway or the 2-C-methyl-D-erythritol-4-phosphate (MEP) pathway [2]. In the MVA pathway, two molecules of acetyl-CoA are formed to acetoacetyl-CoA by acetyl-CoA C-acetyltransferase (AACT), firstly. Then, 3-hydroxy-3-methylglutaryl-CoA (HMG-CoA) is synthesized through adding another acetyl-CoA by 3-hydroxy-3-methylglutaryl-CoA synthase (HMGS). HMG-CoA is further reduced to MVA by 3-hydroxy-3-methylglutaryl-CoA reductase (HMGR). Subsequently, MVA is catalyzed in turn by mevalonate kinase (MK), 5-phosphomevalonate kinase (PMK) and MVAPP decarboxylase (MDC) to generate key intermediate IPP. IPP can be transformed to another precursor DMAPP by isopentenyl-diphosphate deltaisomerase (IPPI). In the MEP pathway, the initial reactants pyruvate and glyceraldehyde 3-phosphate (GA-3P) are catalyzed by 1-deoxy-D-xylulose 5-phosphate synthase (DXS) to form 1-deoxy-D-xylulose 5-phosphate (DXP). Then, DXP is reduced to MEP catalyzed by DXP reductoisomerase (DXR). In the rest of the reactions, there are five enzymes contributing to IPP and DMAPP synthesis, including 2-C-methyl-D-erythritol-4-phosphate cytidyl transferase (MCT), 4-(cytidine 5-diphospho)-2-C-methyl-D-erythritol kinase (CMK), 2-C-methyl-D-erythritol-2,4-cyclodiphosphate synthase (MDS), 1-hydroxy-2-methyl-2-(*E*)-butenyl-4-diphosphate (HMBPP) synthase (HDS), and HMBPP reductase (HDR) [2,3].

In the subsequent cyclization reactions, IPP and DMAPP are transformed into ferruginol catalyzed in turn by copalyl diphosphate synthase (CPS), kaurene synthase-like (KSL) and cytochrome P450 monooxygenase (CYP76AH1). During the last stage, ferruginol is eventually transformed into different tanshinones through some undefined reactions [4].

Recently, researchers have improved the content of tanshinones in *S. miltiorrhiza* through various strategies, including elicitor treatment, hormone signal regulation, overexpression of key biosynthesis genes, and transcriptional regulation [3,5,6]. Nevertheless, few studies have illustrated the role of Ca^2+^ signaling in tanshinone biosynthesis. In plant cells, calcium acts not only as an essential nutrient, but also as a crucial second messenger. When confronted with diverse abiotic and biotic stresses, plant cells generate a cytoplasmic Ca^2+^ signal, which can be decoded by calcium sensors such as calmodulin (CaM), calmodulin-like proteins (CMLs), Ca^2+^-dependent protein kinases (CDPKs), and calcineurin B-like proteins (CBLs) to regulate numerous downstream metabolic reactions [7,8].

Notably, Ca^2+^ signaling is closely related to secondary metabolism in plants [9,10,11,12]. For instance, through binding with a Ca^2+^-CaM complex, transcription factor CAMTA3 promotes the production of glucosinolates, which are defensive compounds against herbivores [9,10]. The biosynthesis of salicylic acid (SA) is controlled by CaM-binding transcription factors such as CBP60g, SARD1 and CAMTA3 [11,12]. Our previous studies have shown that Ca^2+^ signaling is essential to the SA-induced rosmarinic acid accumulation in *S. miltiorrhiza* [13]. Therefore, Ca^2+^ signaling can act as a vital player to regulate secondary metabolite biosynthesis.

Here, we focused on the regulation of secondary metabolism by Ca^2+^ signaling. In this study, bacterial endotoxin lipopolysaccharide (LPS), which is a characteristic glycolipid component of a Gram-negative bacteria cell wall and is a stimulator of pathogen-associated molecular pattern (PAMP) triggered immunity (PTI) [14,15,16], was utilized as an elicitor to treat the wild type hairy roots of *S. miltiorrhiza*. We found that LPS treatment significantly upregulated the expression of key tanshinone biosynthesis genes and enhanced the accumulation of tanshinones in hairy roots. Due to LPS-triggered PTI depending on Ca^2+^ signaling in plant cells [15], the Ca^2+^ channel blocker LaCl_3_ and CaM antagonist W-7 were also used to analyze the role of Ca^2+^ signaling in tanshinone biosynthesis. These results demonstrate that LPS enhances tanshinone biosynthesis in a Ca^2+^-dependent manner and suggest that Ca^2+^ signal transduction is essential for modulating secondary metabolism in *S. miltiorrhiza*.

## 2. Results

### 2.1. LPS Enhances Tanshinone Accumulation in the Wild Type Hairy Roots of S. miltiorrhiza

**Figure 1:**
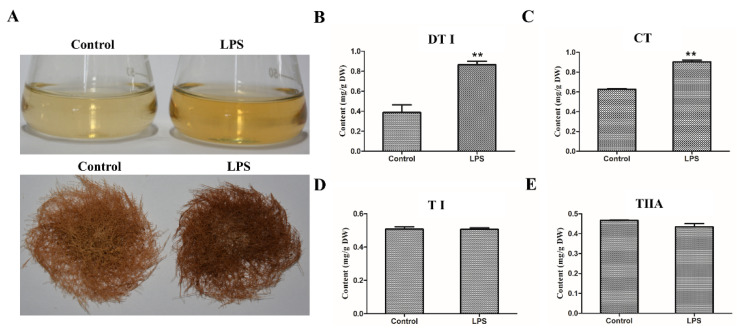
Lipopolysaccharide (LPS) enhances tanshinone accumulation in the wild type (WT) hairy roots of *S. miltiorrhiza*. (**A**) LPS-treated WT hairy roots of *S. miltiorrhiza.* The hairy roots were treated by 50 μg/mL LPS for 10 days. H_2_O was used as a control. (**B**–**E**) The content of tanshinones in LPS-treated hairy roots. The content of the tanshinones was analyzed by HPLC and presented by the means ± SD. The significant differences between different groups were calculated by the Student’s *t*-test. (**) indicates a very significant difference (*p* ≤ 0.01). TI, tanshinone I; TIIA, tanshinone IIA; CT, cryptotanshinone; DT, dihydrotanshinone.

Since the biosynthesis of secondary metabolites might be induced by microorganisms, the bacterial component LPS was applied as a novel elicitor to treat the wild type (WT) hairy roots of *S. miltiorrhiza*. After being treated by 50 μg/mL LPS for 10 days, the hairy roots and the culture medium showed a deep red color, which is the characteristic color of tanshinones (Figure 1A). Compared to the control, LPS did not obviously affect the growth of hairy roots (Figure 1A). Further, the content of the tanshinones, including dihydrotanshinone I (DTI), cryptotanshinone (CT), tanshinones I (TI) and tanshinones IIA (TIIA), was analyzed by HPLC. When the hairy roots were treated by LPS, the content of DTI significantly increased from 0.38 mg/g to 0.86 mg/g in contrast to the control (Figure 1B), and the content of CT increased from 0.62 mg/g to 0.9 mg/g (Figure 1C). However, the content of TI and TIIA showed no significant change (Figure 1D,E). These results indicate that LPS can induce the accumulation of DTI and CT without markedly inhibiting the growth of *S. miltiorrhiza* WT hairy roots.

### 2.2. LPS Upregulates Key Gene’s Expression in Tanshinone Biosynthesis Pathways

**Figure 2:**
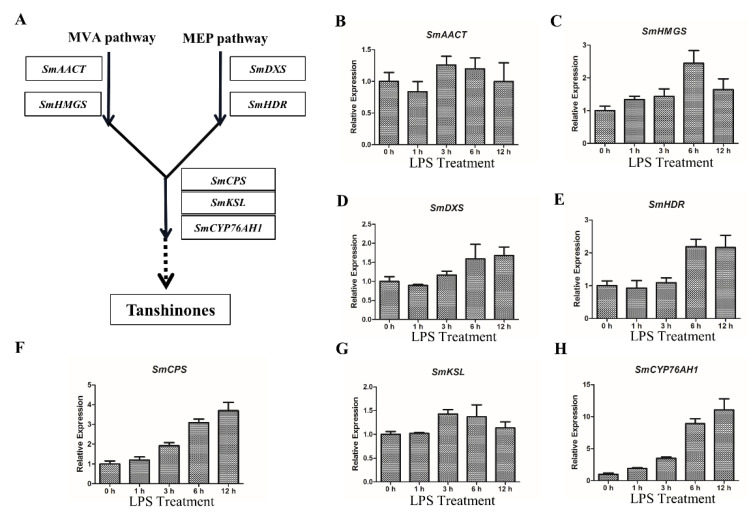
LPS upregulates the expression of key tanshinone biosynthesis genes in the WT hairy roots of *S. miltiorrhiza*. (**A**) The biosynthesis pathways of tanshinones in *S. miltiorrhiza*. (**B**,**C**) The relative expression levels of key genes in the MVA pathway. The transcript levels of *SmAACT* and *SmHMGS* were analyzed by qRT-PCR using *SmACT* for normalization. (**D**,**E**) The relative expression levels of key genes in the MEP pathway. The transcript levels of *SmDXS* and *SmHDR* were analyzed by qRT-PCR using *SmACT* for normalization. (**F**–**H**) The relative expression levels of key downstream genes in tanshinone biosynthesis. The transcripts levels of *SmCPS*, *SmKSL* and *SmCYP76AH1* were analyzed by qRT-PCR using *SmACT* for normalization. (**B**–**H**) The hairy roots were treated by 50 μg/mL LPS in a time gradient. The gene’s expression level of 0 h was set to 1. The expression value of genes is shown as the means ± SD.

To elucidate the regulation mechanism of LPS, the key biosynthesis genes of tanshinones were analyzed by qRT-PCR. In the biosynthesis pathways of tanshinones, *SmAACT* and *SmHMGS* are from the MVA pathway, and *SmDXS* and *SmHDR* are from the MEP pathway (Figure 2A). After being induced by LPS, the transcripts levels of *SmAACT* and *SmHMGS* were obviously upregulated at 6 h (Figure 2B,C). Similarly, *SmDXS* and *SmHDR* showed the same response to LPS treatment (Figure 2D,E). In the confirmed biosynthesis pathway of tanshinones, *SmCPS*, *SmKSL* and *SmCYP76AH1* are located downstream the MVA and MEP pathways (Figure 2A). Notably, the expression of these three genes also increased along with LPS treatment (Figure 2F–H). *SmCPS* and *SmCYP76AH1* were especially upregulated by LPS in a time-dependent manner (Figure 2F,H).

**Figure 3:**
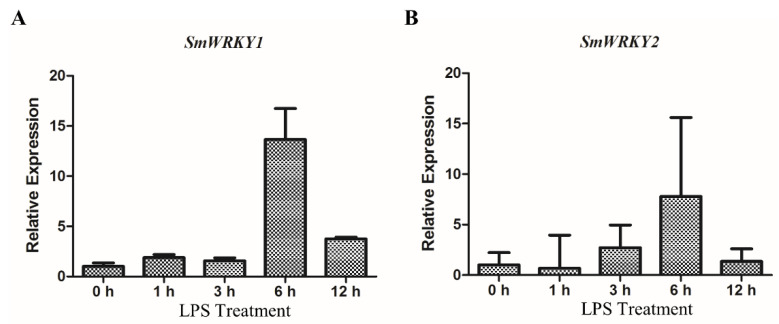
LPS upregulates the expression of *SmWRKY1* and *SmWRKY2* in the WT hairy roots of *S. miltiorrhiza*. (**A**,**B**) The relative expression levels of *SmWRKY1* and *SmWRKY2* were analyzed by qRT-PCR using *SmACT* for normalization. The hairy roots were treated by 50 μg/mL LPS in a time gradient. The gene’s expression level of 0 h was set to 1. The expression value of genes is shown as the means ± SD.

To further explore the stimulation mechanism of these biosynthesis genes by LPS, the expression of transcription factors *SmWRKY1* and *SmWRKY2* was analyzed by qRT-PCR. In *S. miltiorrhiza*, *SmWRKY1* can bind with the promoter of *SmDXR*, and *SmWRKY2* can bind with *SmDXS* and *SmCPS,* to positively regulate tanshinone biosynthesis [5,17,18]. Our further analysis indicated that LPS upregulated the transcript levels of *SmWRKY1* and *SmWRKY2* in the same time-dependent manner. The expression of these transcription factors responded to LPS and reached a peak at 6 h (Figure 3A,B). Hence, *SmDXS, SmCPS* and *SmDXR* can be highly transcribed due to the activation of *Sm*WRKY1 and *Sm*WRKY2.

Taken together, the secondary metabolite tanshinones can be induced by the immune regulator LPS. LPS enhances tanshinone accumulation through stimulating *Sm*WRKY1- and *Sm*WRKY2-regulated gene expression in tanshinone biosynthesis pathways.

### 2.3. Ca^2+^ Inhibitors Affect Tanshinone Accumulation

**Figure 4:**
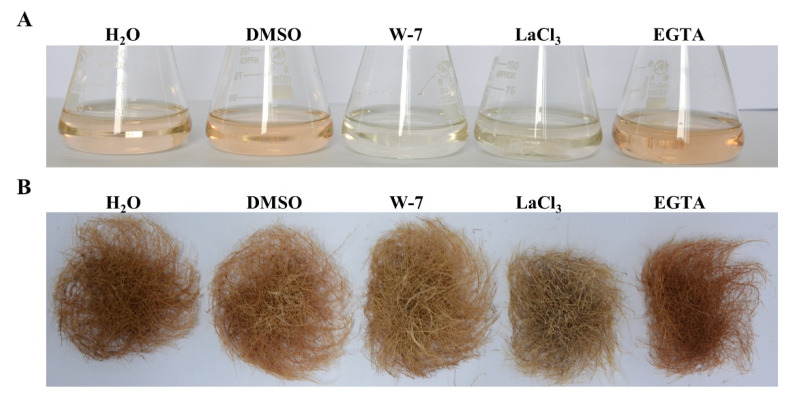
Ca^2+^ inhibitors affect tanshinone accumulation in the WT hairy roots of *S. miltiorrhiza*. (**A**) The culture medium of different treated hairy roots. (**B**) The different treated hairy roots. (**A**,**B**) The hairy roots were treated by 1 mmol/L LaCl_3_, 100 μmol/L W-7 and 1mmol/L EGTA for 10 days, respectively. H_2_O was the control of LaCl_3_ and EGTA. DMSO was the control of W-7.

Ca^2+^ signal transduction is essential for the LPS-triggered plant immune response [15]. Thus, three Ca^2+^ signal inhibitors, including Ca^2+^ channel blocker LaCl_3_, CaM antagonist W-7 and Ca^2+^ chelator EGTA, were applied to analyze the role of Ca^2+^ signaling in tanshinone biosynthesis [15,19]. Since tanshinones can generate a deep red color in the roots of *S. miltiorrhiza*, we preliminarily observed the color of the hairy roots treated by different Ca^2+^ reagents. As shown in Figure 4A,B, the hairy roots treated by 1 mmol/L LaCl_3_ apparently showed a light color compared to the H_2_O control. Similarly, 100 μmol/L W-7 also led to light color in contrast to the DMSO control. Nevertheless, 1mmol/L EGTA did not obviously affect the color of the hairy roots compared to the H_2_O control. These results suggest that Ca^2+^ signaling is closely associated with tanshinone biosynthesis. The accumulation of tanshinones might be inhibited by blocking Ca^2+^ influx or repressing CaM-mediated signaling in the hairy roots of *S. miltiorrhiza*.

### 2.4. Ca^2+^ Channel Blocker Inhibits LPS-Induced Tanshinone Accumulation

**Figure 5:**
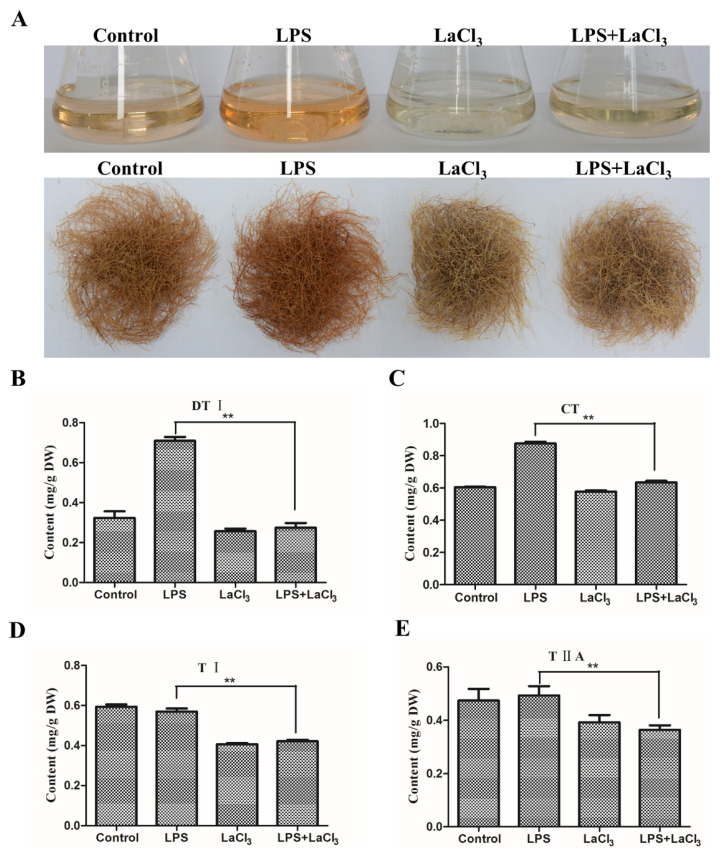
LaCl_3_ affects tanshinone accumulation in the WT hairy roots of *S. miltiorrhiza*. (**A**) The cultures of treated hairy roots. The WT hairy roots were treated by 50 μg/mL LPS, 1 mmol/L LaCl_3_ and 50 μg/mL LPS + 1 mmol/L LaCl_3_ for 10 days. H_2_O was used as a control. (**B**–**E**) The content of tanshinones in treated hairy roots. The content of tanshinones in H_2_O, LPS, LaCl_3_ and LPS+LaCl_3_ treated hairy roots was analyzed by HPLC. The bars are shown as the means ± SD. The significant differences between different groups were calculated by the Student’s *t*-test. (**) indicates a very significant difference (*p* ≤ 0.01).

LaCl_3_ is capable of suppressing cytoplasmic Ca^2+^ elevation via blocking Ca^2+^ influx [15,19]. Thus, LaCl_3_ was synergistically utilized with LPS to analyze the role of Ca^2+^ signaling in tanshinone biosynthesis. As shown in Figure 5A, the LPS-treated hairy roots showed the deepest color and LaCl_3_ treatment resulted in the lightest color. LPS-induced deep red was apparently decreased by LaCl_3_ synergetic treatment (Figure 5A). Further, the content of tanshinones was examined by HPLC. Compared to the LPS treatment, the content of DTI in the LaCl_3_+LPS-treated sample significantly reduced from 0.71 mg/g to 0.28 mg/g, and CT reduced from 0.88 mg/g to 0.63 mg/g (Figure 5B,C). The LPS+LaCl_3_ treatment also led to a significant reduction in TI and TIIA in a similar way (Figure 5D,E). These results confirmed that with the inhibition of Ca^2+^ influx by LaCl_3_, LPS-induced tanshinone accumulation was accordingly diminished. Therefore, the Ca^2+^ influx signal is involved in regulating tanshinone accumulation.

### 2.5. CaM Antagonist Inhibits LPS-Induced Tanshinone Accumulation

**Figure 6:**
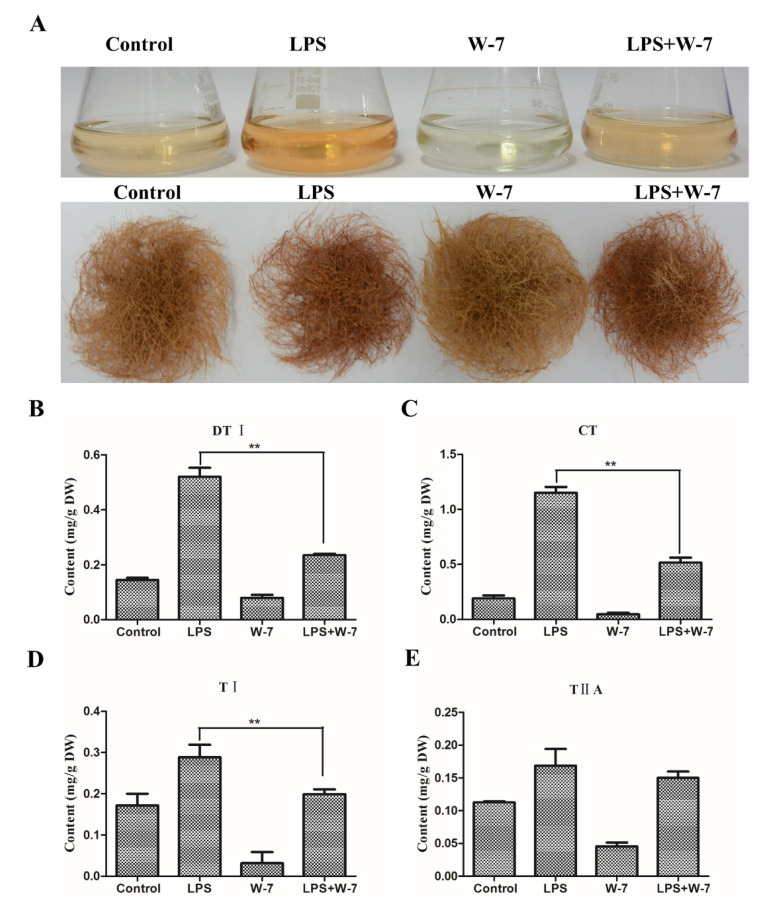
W-7 affects tanshinone accumulation in the WT hairy roots of *S. miltiorrhiza*. (**A**) The cultures of treated hairy roots. The WT hairy roots were treated by 50 μg/mL LPS, 100 μmol/L W-7 and 50 μg/mL LPS + 100 μmol/L W-7 for 10 days, DMSO was used as a control. (**B**–**E**) The content of tanshinones in treated hairy roots. The content of tanshinones in DMSO, LPS, W-7 and LPS+W-7 treated hairy roots was analyzed by HPLC. The bars are shown as the means ± SD. The significant differences between different groups were calculated by the Student’s *t*-test. (**) indicates a very significant difference (*p* ≤ 0.01).

**Figure 7:**
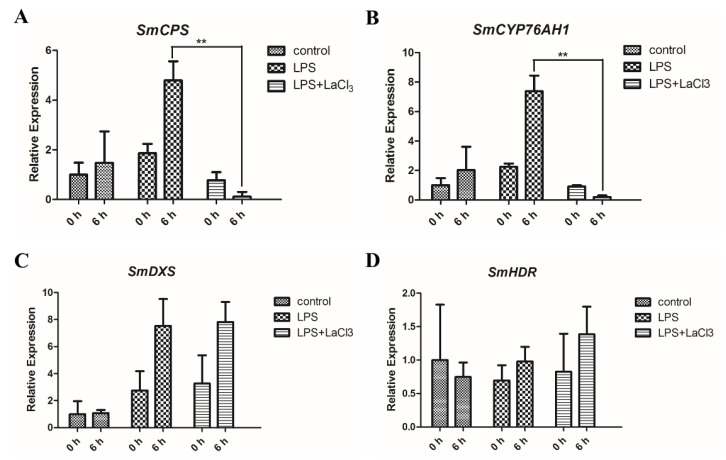
LaCl_3_ regulates the expression of downstream tanshinone biosynthesis genes. (**A**,**B**) The expression of *SmCPS* and *SmCYP76AH1* in treated hairy roots. The WT hairy roots were treated by 50 μg/mL LPS and 50 μg/mL LPS + 1 mmol/L LaCl_3_ for 6 h. H_2_O was used as a control. *SmCPS* and *SmCYP76AH1* were analyzed by qRT-PCR using *SmACT* for normalization. The expression value of genes is shown as the means ± SD. (**C**,**D**) The expression of *SmDXS* and *SmHDR* in treated hairy roots. The expression of *SmDXS* and *SmHDR* were analyzed by qRT-PCR using *SmACT* for normalization. (**A**–**D**) The gene’s expression level of 0 h was set to 1. The expression value of genes is shown as the means ± SD. The significant differences between different groups were calculated by the Student’s *t*-test. (**) indicates a very significant difference (*p* ≤ 0.01).

CaM serves as a crucial sensor in Ca^2+^ signal transduction. Through binding with Ca^2+^, the Ca^2+^-CaM complex interacts with target proteins such as CNGC, CDPK, and MAPK to regulate numerous metabolism reactions [8,20]. Hence, the CaM antagonist W-7 was utilized to corporately treat hairy roots with LPS. As shown in Figure 6A, W-7 treatment partly decreased the LPS-induced deep red of the hairy roots and generated the lightest color, while showing no obvious growth inhibition. Compared to LPS treatment, the content of DTI, CT and TI significantly declined from 0.52 mg/g to 0.23 mg/g, 1.15 mg/g to 0.52 mg/g, and 0.29 mg/g to 0.20 mg/g in LPS+W-7 treated hairy roots (Figure 6B–D). Notably, the separate W-7 treatment resulted in extreme inhibition of these four tanshinones, especially CT and TI (Figure 6B–E). Taken together, CaM-mediated signaling is essential for LPS-induced tanshinone accumulation in *S. miltiorrhiza* hairy roots.

### 2.6. LPS Induces the Expression of Key Tanshinone Biosynthesis Genes in a Ca^2+^-Dependent Manner

To further investigate the role of Ca^2+^ signaling in tanshinone biosynthesis, the expression levels of key genes in LPS and LaCl_3_ treated WT hairy roots were analyzed by qRT-PCR. When synergistically treated by LaCl_3_ and LPS for 6 h, the expression levels of *SmCPS* and *SmCYP76AH1* reduced approximately 40-fold and 37-fold, respectively, compared to the LPS separate treatment (Figure 7A,B). However, *SmHDR* and *SmDXS* did not show apparent reduction by LaCl_3_+LPS treatment (Figure 7C,D). Comparatively, LaCl_3_ preferentially inhibits the downstream genes (*SmCPS* and *SmCYP76AH1*) in tanshinone biosynthesis pathways. This suggests that the downstream genes of the tanshinone biosynthesis pathway are more likely to be regulated by Ca^2+^ signaling than the MEP pathway genes.

**Figure 8:**
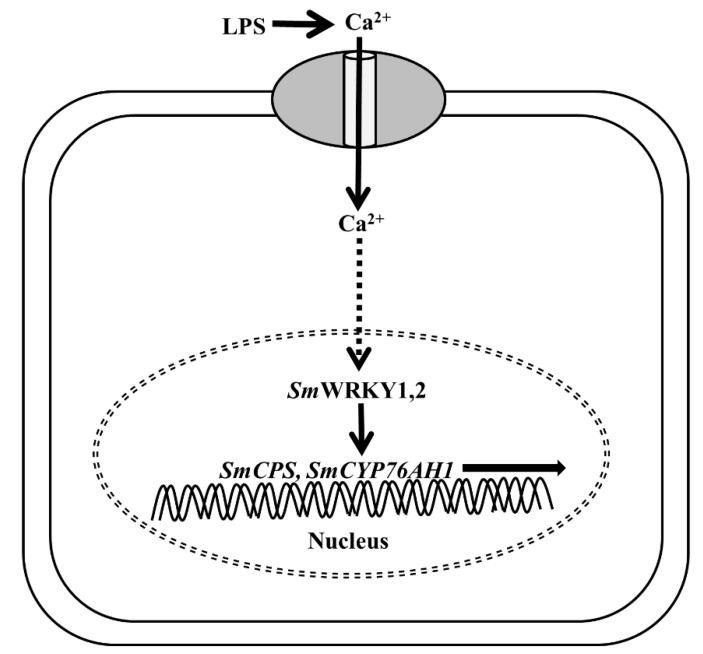
The diagram of LPS-induced tanshinone biosynthesis in *S. miltiorrhiza.* LPS induces tanshinone biosynthesis in a Ca^2+^-dependent manner. Firstly, LPS treatment leads to a Ca^2+^ elevation signal in the cytoplasm. Then, the transcription factors *Sm*WRKY1 and *Sm*WRKY2 are activated by some undetermined Ca^2+^-related regulators. Finally, key tanshinone biosynthesis genes are upregulated by *Sm*WRKY1 and *Sm*WRKY2 resulting in tanshinone accumulation in hairy roots of *S. miltiorrhiza.*

Therefore, we present the mechanism of LPS-induced tanshinone biosynthesis in Figure 8. Firstly, LPS induces the generation of Ca^2+^ signaling in the cytoplasm, which is accordingly decoded by the Ca^2+^-dependent regulators. Then, *SmWRKY1* and *SmWRKY2* are upregulated and activated by some undefined Ca^2+^-dependent regulators. Eventually, the key biosynthesis genes of tanshinones such as *SmCPS*, *SmDXS*, *SmDXR* and *SmCYP76AH1* are transcribed in a high level that in turn synthesizes the tanshinones in the hairy roots of *S. miltiorrhiza*.

## 3. Discussion

The dry roots of *S. miltiorrhiza* (Danshen) have been used in Traditional Chinese Medicine (TCM) since 200–300AD [2]. Because of slow growth and a low content of bioactive components, the wild resources of *S. miltiorrhiza* cannot meet the growing requirements from pharmaceutical markets. Therefore, improving the content of pharmacological ingredients is the main purpose of metabolic research. Up to now, many approaches have been applied to enhance the content of phenolic acids and tanshinones in *S. miltiorrhiza* [21]. In this study, the bacterial component lipopolysaccharide (LPS) was utilized as a novel elicitor to induce the wild type hairy roots of *S. miltiorrhiza*. According to the biosynthesis pathway of tanshinones, cryptotanshinone (CT) is the first tanshinone to be generated, and then tanshinone IIA (TIIA), tanshinone IIB (TIIB), tanshinone I (TI), and dihydrotanshinone I (DTI) [3,22]. We have found that LPS significantly enhances the accumulation of tanshinones CT and DTI. Furthermore, the gene expression analysis has shown that key genes from the MVA pathway (*SmAACT*, *SmHMGS*), the MEP pathway (*SmDXS*, *smHDR*) and the downstream biosynthesis pathway (*SmCPS*, *SmKSL*, *SmCYP76AH1*) respond to LPS treatment in a time-dependent manner. These results demonstrate that LPS is capable of activating key genes’ expression in the tanshinone biosynthesis process. It is worth noting that LPS does not obviously inhibit the growth of hairy roots. Thus, LPS can be applied as a positive elicitor to enhance the content of tanshinones without affecting the growth of the *S. miltiorrhiza* hairy roots. This is valuable for increasing the content of metabolites.

In *S. miltiorrhiza*, researchers have promoted the content of tanshinones via pathway engineering such as *SmGGPPS-SmDXS2* [23], *SmHMGR-SmDXR* [24]; or overexpression of key transcription factors including *SmMYB98* [25], *SmMYB36* [26], *SmWRKY1* [17], *SmWRKY2* [5], and *SmbHLH3* [6]. Previous studies have confirmed that *SmDXS2* and *SmCPS* are the target genes of *SmWRKY2* [5], and *SmDXR* is the target of *SmWRKY1* [17]. Our results have further shown that *SmWRKY1* and *SmWRKY2* were upregulated by LPS in the same manner as *SmCPS, SmDXS2, SmCYP76AH1*, etc. Therefore, we deduce that LPS promotes tanshinone biosynthesis through activating the *SmWRKY1,2*-*SmCPS, SmDXS2, SmDXR* pathway. In plants, WRKY transcription factors act as a key regulator in response to diverse abiotic and biotic stresses [18,27], including PAMP triggered immunity (PTI) [28,29]. WRKYs can interact with Ca^2+^-related regulators to regulate immune reactions, such as CPK and calmodulin [30,31,32,33]. These suggest that tanshinone metabolism is closely related with Ca^2+^ signaling and might be regulated in a similar way by immunity responding reactions.

The bacterial component LPS is an immune activator. It is capable of inducing cytoplasm Ca^2+^ elevation, which is essential for the plant innate immune response [15,34]. To analyze the role of Ca^2+^ signaling in tanshinone biosynthesis, *S. miltiorrhiza* hairy roots were collaboratively treated by LPS and Ca^2+^ inhibitors. Both Ca^2+^ channel blocker LaCl_3_ and CaM antagonist W-7 can significantly inhibit the accumulation of tanshinones. Further analysis has shown that the downstream biosynthetic genes (*SmCPS*, *SmCYP76AH1*) are presumably regulated by Ca^2+^ signaling in priority. Based on these data, we present the pathway of LPS-induced tanshinone biosynthesis as Ca^2+^ signal-Ca^2+^-dependent regulators-*Sm*WRKY1,2-downstream genes axis in *S. miltiorrhiza*. Our study provides a new insight into the essential role of Ca^2+^ signaling in tanshinone biosynthesis. However, the exact mechanism of how *SmWRKY1* and *SmWRKY2* are modulated by Ca^2+^-dependent regulators remains unresolved. In the future, searching for the Ca^2+^-dependent master regulators, which are capable of activating *SmWRKY1* and *SmWRKY2*, might be the key to uncovering the mechanism of Ca^2+^-mediated tanshinone biosynthesis in *S. miltiorrhiza.* For the purpose of promoting the content of valuable metabolites, the Ca^2+^ transduction pathway might be the potential regulation target.

Based on our findings, the LPS-induced Ca^2+^ signal is highly associated with ion influx sourced from apoplast. In plant tissues, the arabinogalactan proteins (AGPs), negatively charged and anchored to the extracellular side of the plasma membrane, can reversibly bind with Ca^2+^ and hypothetically serve as the calcium capacitor [35]. Triggered by a low pH related to plasma membrane (PM) H^+^-ATPases, the AGPs-Ca^2+^ complex can release free Ca^2+^ into the cell-surface apoplast and in turn lead to [Ca^2+^]_cyt_ signal generation [35]. In recent in-depth research, knockouts of the key β-glucuronosyltransferases (GlcATs), which are responsible for adding glucuronic acid (GlcA) to AGPs, resulted in reduced AGPs glucuronidation, impaired Ca^2+^ signaling and consequent deficient plant development [36,37]. This AGPs-Ca^2+^ interaction model highlights the crucial role of the proton pump in modulating Ca^2+^ signaling. The post-translational regulation, especially phosphorylation, is central to alternating PM H^+^-ATPases between the auto-inhibited state and active state [38]. For instance, fusicoccin, the secreta of fungi *Fusicoccum amygdali*, is able to activate plant PM H^+^-ATPases by increasing the phosphorylation level [38,39]. Notably, LPS-induced phosphorylation of key proteins such as AMPK [40], p53 [41] and mTOR [42], has been proved by massive studies in animals. In plants, LPS might similarly regulate phosphorylation of crucial proteins and might be a potential activator of PM H^+^-ATPases. Thus, we further hypothesize that LPS induces Ca^2+^ influx via the regulation of PM H^+^-ATPases. The phosphorylation modification of PM H^+^-ATPases could be the key to uncover LPS-generated Ca^2+^ influx in *S. miltiorrhiza.*

In addition, in comparison with the elaborated studies in animals, LPS-regulated pathways in plants are still elusive. Up to date, several proteins including *At*LBR1,2 (LPS binding protein) and *Os*CERK1 (LysM-type receptor-like kinase) have been determined as the key players in LPS-induced immune responses [43,44]. However, the potential correlations between these LPS-related regulators and secondary metabolism have not been deeply elucidated. Consequently, the regulatory network of LPS in plants still needs to be illuminated by more comprehensive research in the future.

## 4. Materials and Methods

### 4.1. Reagents

Lipopolysaccharides (L9143) and LaCl_3_ (449830) were from Sigma-Aldrich (St. Louis, MO, USA) and were dissolved in sterile water. W-7 (N-(6-Aminohexyl)-5-chloro-1-naphthalenesulfonamide Hydrochloride) (N136431) was from Aladdin (Shanghai, China) and was dissolved in DMSO. The SteadyPure Plant RNA Extraction Kit (AG21019), the Evo M-MLV RT Kit with gDNA Clean for qPCR (AG11601), and the SYBR^®^ Green Premix Pro Taq HS qPCR Kit (AG11701) were from Accurate Biotechnology(Changsha, China). Acetonitrile and methyl alcohol for HPLC analysis were from TEDIA (Fairfield, OH, USA). The standards of DT, CT, TI and TIIA were from Herbpurify (Chengdu, China).

### 4.2. Hairy Roots Culture and Treatment

The *S. miltiorrhiza* wild type (WT) hairy roots were generated by *Agrobacterium rhizogenes* (ATCC15834). The generation and culture of hairy roots were based on previous research [45]. Before analysis, hairy roots weighing 0.3 g were cultured in 50 mL 6, 7-V liquid medium [46] containing an amount of 30 g/L sucrose for 21 days at 25 °C. LPS and other reagents were added into the culturing medium on the 10th day, and then the hairy roots were harvested on the 21st day. The hairy roots were dried at 45 °C for 4 days before HPLC analysis.

### 4.3. Reverse Transcription and Quantitative Real-Time PCR Analysis

To investigate the expression of key biosynthesis genes, the WT hairy roots were treated by 50 μg/mL LPS in a time gradient, and the 0 h treatment was used as a control. The total RNAs of the control and LPS-treated hairy roots were extracted by a SteadyPure Plant RNA Extraction Kit (AG21019). Total RNA (1 μg) was reversely transcribed by an Evo M-MLV RT Kit (with gDNA Clean) (AG11601). The gene expression was analyzed by a SYBR^®^ Green Premix Pro Taq HS qPCR Kit (AG11701). qRT-PCR was conducted on a real-time PCR system (Bio-RAD CFX96, Hercules, CA, USA). *SmACT* was used as a reference gene. The relative expression level of a gene was calculated by the 2^−ΔΔCt^ method. The gene’s expression level of 0 h was set to 1. Gene-specific primers were shown in the Appendix A Table A1.

To investigate the regulation of Ca^2+^ signaling in gene expression, the WT hairy roots were treated by 50 μg/mL LPS and 50 μg/mL LPS + 1 mmol/L LaCl_3_ for 6 h, and H_2_O was used as a control. The total RNAs of the control and the different treated hairy roots were extracted, reversely transcribed and analyzed by qRT-PCR, as mentioned above. The gene’s expression level of control at 0 h was set to 1.

### 4.4. HPLC Analysis

The dried hairy roots powder (0.02 g) was extracted by 70% methyl alcohol (4 mL) overnight and treated by ultrasonic for 45 min. Then, the mixture was centrifuged at 10,000 rpm for 10 min. The supernatant was filtered through a 0.45 µm membrane before HPLC analysis. The content of DTI, CT, TI and TIIA was determined by the Waters 1525/2489 HPLC system (Milford, MA, USA) equipped with an InertSustain^®^ C18 column (5 um, 250 mm × 4.6 mm, SHIMADZU-GL, Tokyo, Japan).

The HPLC operation software was Empower 2. The detection wavelength for tanshinones was 270 nm. Elution gradients are as follows (A: acetonitrile; B: 0.02% phosphoric acid solution): 0–10 min, 5–20% A; 10–15 min, 20–22% A; 15–20 min, 22–25% A; 20–28 min, 25–30% A; 28–40 min, 30–35% A; 40–45 min, 35–45% A; 45–50 min, 45–50% A; 50–58 min, 50–58% A; 58–67 min, 58–50% A; 67–70 min, 50–60% A; 70–80 min, 60–70% A; 80–85 min, 70–100% A; 85–95 min, 100–5% A. The column temperature was set at 30 °C. The flow rate was set as 1 mL/min.

### 4.5. Statistical Analysis

All experiments were conducted over three times. The results were described as the mean ± standard deviation (SD). The significant differences between different groups were calculated by the Student’s *t*-test. (*) indicates a significant difference (0.01 < *p* < 0.05). (**) indicates a very significant difference (*p* ≤ 0.01).

## 5. Conclusions

On the basis of the data in this study, we proposed the model of LPS-enhanced tanshinone biosynthesis in *S. miltiorrhiza*. Firstly, LPS induces a cytoplasmic Ca^2+^ signal which consequently activates the expression of the transcription factors *SmWRKY1* and *SmWRKY2* via some undefined Ca^2+^ sensors. Then, the key biosynthesis genes of tanshinones are upregulated by *Sm*WRKY1 and *Sm*WRKY2. To summarize, the Ca^2+^ signal-Ca^2+^-dependent regulators-*Sm*WRKY1,2-downstream genes axis might be central to regulate tanshinone biosynthesis in *S. miltiorrhiza*.

## Abbreviations

LPS	lipopolysaccharide
TI	tanshinone I
TIIA	tanshinone IIA
TIIB	tanshinone IIB
CT	cryptotanshinone
DTI	dihydrotanshinone I

## Appendix A

**Table A1:** Primers for qRT-PCR.

Primer	Sequence (5′–3′)
*SmACT-*F	GGTGCCCTGAGGTCCTGTT
*SmACT-*R	AGGAACCACCGATCCAGACA
*SmAACT1-*F	TGAAGGACGGACTCTGGGATGT
*SmAACT1-*R	CCTTGTCAACAATGGTGGATGG
*SmCPS1-*F	CCACATCGCCTTCAGGGAAGAAAT
*SmCPS1-*R	TTTATGCTCGATTTCGCTGCGATCT
*SmCYP76AH1-*F	ACGCATCACTTCACCCATCTCA
*SmCYP76AH1-*R	ATTGCCGACTCATCCACGAT
*SmDXS2-*F	CTCACGGTCGCATTGCATCAT
*SmDXS2-*R	CGCTTTCGTCTCGTTTAGGGA
*SmHDR1-*F	GGATTTGACCCGGACAAGGAT
*SmHDR1-*R	CCGCCAATGACTAGGATGAGA
*SmHMGS1-*F	TTAGGGCGAATCACATGGCTCA
*SmHMGS1-*R	TCGGCATCCAAGATCGAGAAC
*SmKSL1-*F	TGGAAACAGTGTGACCCTTCTGCT
*SmKSL1-*R	GCTTGCATACAAATAACACCCAATCCT
*SmWRKY1-*F	ACCTACAACGGCCAACACACT
*SmWRKY1-*R	TCGTCCGGTGTTTTCATTTG
*SmWRKY2-*F	ACTCATCCAAGCTGTCCGGT
*SmWRKY2-*R	ATTCATTGTTCCGTTTGAGCC
